# *Helicobacter pylori* eradication improves the quality of life regardless of the treatment outcome: A multicenter prospective cohort study

**DOI:** 10.1097/MD.0000000000009507

**Published:** 2017-12-29

**Authors:** Hiroki Taguchi, Shuji Kanmura, Takuro Maeda, Hiromichi Iwaya, Shiho Arima, Fumisato Sasaki, Yuichirou Nasu, Shiroh Tanoue, Shinichi Hashimoto, Akio Ido

**Affiliations:** Digestive and Lifestyle Diseases, Kagoshima University Graduate School of Medical and Dental Sciences, Kagoshima, Japan.

**Keywords:** dyspepsia, epigastralgia, eradication treatment, *Helicobacter pylori*, mental component summary, physical component summary, quality of life

## Abstract

*Helicobacter pylori* (Hp) eradication is recommended for improving the quality of life (QOL) of patients with epigastric symptoms, especially reflux and dyspepsia. However, no reports have investigated the improvement of QOL after the eradication of Hp irrespective of epigastric symptoms. The aim of this study was to investigate the improvement in the QOL after the eradication of Hp irrespective of epigastric symptoms, and evaluate the factors associated with an improved QOL after the eradication of Hp.

This prospective cohort study was performed at 15 referral institutions from September 2013 to December 2014. The patients’ QOL and epigastric symptoms were evaluated before and after the eradication of Hp using the 8-item Short-Form Health Survey (SF-8) and the modified Frequency Scale for the Symptoms of Gastroesophageal Reflux Disease, respectively.

One hundred sixty-five of 184 Hp-infected patients underwent Hp eradication treatment. The treatment was successful in 82.4% (136/165) of the cases. One hundred sixty of the 165 Hp-infected patients were eligible for inclusion in the QOL analysis. In the indices of QOL on the SF-8, the scores on both the mental component summary (MCS) and the physical component summary (PCS) were found to have significantly improved after the eradication of Hp. However, the epigastric symptoms before the eradication of Hp were not correlated with either the MCS or PCS. A low QOL value before the eradication of Hp was the factor what was most strongly associated with the improvement in the QOL.

The eradication of Hp improved the QOL, regardless of the outcome of the treatment, especially in patients who had an impaired QOL before the eradication.

## 1 Introduction

Over 3 decades have passed since the discovery of *Helicobacter pylori* (Hp) in 1983.^[1]^ The involvement of Hp infection in the carcinogenesis of gastric cancer already been recognized; thus the eradication of Hp has been strongly recommended throughout the world.^[2,3]^ In addition, it has shown that Hp infection affects various diseases, including: peptic ulcers, idiopathic thrombocytopenic purpura (ITP), gastric lymphoma of mucosa-associated lymphoid tissue (MALT), iron-deficiency anemia and chronic urticaria.^[4–8]^ In Japan, national health insurance covers Hp eradication therapy for patients who have peptic ulcers, MALT, ITP, atrophic gastritis, and early gastric cancer after endoscopic submucosal dissection. Actually, many patients with Hp infection have various epigastric symptoms, such as gastroesophageal reflux disease (GERD), functional dyspepsia (FD). Hp-infected patients who are asymptomatic^[9]^ may also undergo Hp eradication therapy to prevent gastric carcinogenesis, despite the absence of any gastric symptoms.

Hp infection might subclinically impair various mental and physical aspects of a patient's quality of life (QOL). Several authors have reported that the QOL of patients with epigastric symptoms was improved after the eradication of Hp.^[10,11]^ However, there are no reports that refer to the changes in QOL after the eradication of Hp irrespective of epigastric symptoms and no studies have investigated the QOL after the eradication of Hp in a cohort that includes Hp-infected patients without gastric symptoms. In the present study, we analyzed whether or not Hp eradication improved the QOL and determined the factors associated with an improvement in the QOL after the eradication of Hp in patients with and without epigastric symptoms.

## 2 Methods

### 2.1 Participants

Patients who were endoscopically diagnosed with atrophic gastritis between September 2013 and December 2014 at Kagoshima University Hospital and 14 affiliated hospitals were enrolled in the present study. Patients with peptic ulcers, reflux esophagitis (grade ≥A of the Los Angeles classification^[12]^), prior Hp eradication therapy, severe renal or liver dysfunction, a history of gastric surgery, or who used proton pump inhibitors (PPIs) (regular full use), nonsteroid anti-inflammatory drugs (regular use), antidepressant or antipsychotic drugs, or acotiamide were excluded from the present study. This study was approved by the Kagoshima University Hospital Institutional Review Board (IRB), and the IRBs of the 14 affiliated hospitals. This study was performed in accordance with the declaration of Helsinki. Written informed consent was obtained from all of the patients who participated in the study.

### 2.2 Study protocol

This was a multicenter prospective cohort study (UMIN000013060). The 8-item Short-Form Health Survey (SF-8) and modified Frequency Scale for the Symptoms of Gastroesophageal Reflux Disease (mFSSG) scores of the Hp-positive patients were determined (hereafter, referred to as the “registration point”). If the mFSSG score was 0 points, Hp eradication therapy was performed immediately. However, if the score was >0 points, Hp eradication therapy was performed after the administration of a PPI [Rabeprazole, 10 mg, semel in die (s.i.d.)] for 4 weeks (hereafter, referred to as the “eradication point”). PPIs were withdrawn for a period of 4 to 6 weeks after the eradication of Hp to determine the efficacy of the treatment. In terms of the study protocol, the 4 to 6-week PPI-free period was held for 2 reasons: first, to confirm the eradication of Hp; and second, to provide a washout period for the effects of PPIs in patients with epigastric symptoms.

**Figure 1:**
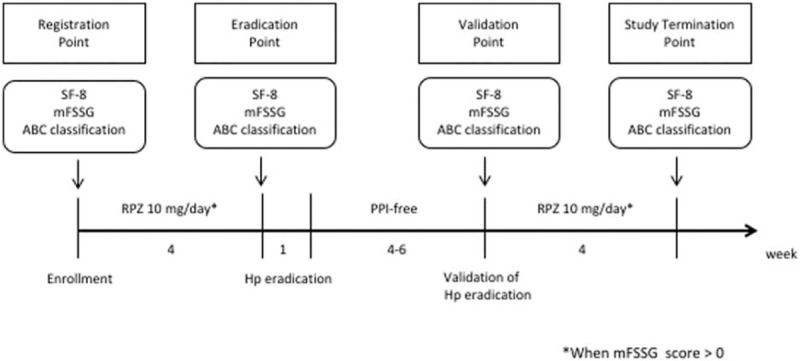
The study protocol. At the “registration point” and “validation point,” the study participants whose mFSSG scores were >0 received rabeprazole (10 mg/d). Meanwhile, those whose mFSSG scores were = 0 did not receive rabeprazole (10 mg/d). Thus, the registration point and validation point (which both occurred prior to the administration of the proton pump inhibitor), were compared to analyze the condition of the patients before and after the eradication of Hp. Hp = *Helicobacter pylori*; mFSSG = modified Frequency Scale for the Symptoms of Gastroesophageal Reflux Disease; PPI = proton pump inhibitors; SF-8 = 8-item Short-Form Health Survey.

The ^13^C-Urea Breath Test (^13^C-UBT) was then performed (hereafter, referred to as the “validation point”) to evaluate the effect of Hp eradication. At the validation point, the patients’ SF-8 and mFSSG scores were determined in the same manner as at the registration point. If the mFSSG score was >0 points, then a PPI (Rabeprazole, 10 mg s.i.d.) was administered for 4 weeks; the time point after the administration of the PPI was referred to as the “study termination point” (Fig. 1). When a patient had an mFSSG score of 0 at the registration point, then his or her score was the same as that at the eradication point. Meanwhile, when a patient's mFSSG score was 0 at the validation point, his or her score at the validation point was the same as that at the study termination point. At the validation point, none of the patients were informed of whether the eradication had been successful to avoid a selection bias. The primary endpoint was the improvement in the QOL after the eradication of Hp. In addition, the serum levels of pepsinogen and Hp immunoglobulin G (IgG) antibodies were evaluated before and after the eradication of Hp.

### 2.3 SF-8

The patients’ QOL was evaluated using the SF-8 [*Manual of the SF-8 Japanese version,* Japanese translation.^[13,14]^ The SF-8 consists of 8 questions in the following fields (“general health,” “physical function,” “physical role,” “bodily pain,” “vitality,” “social function,” “mental health,” and “emotional role”). The respondent must provide 1 answer for each of the 8 questions. The scores for 2 summaries (the physical component summary [PCS] and the mental component summary [MCS]) are calculated. These summaries are evaluated as indices of the QOL. The mean scores for each of the summaries in the Japanese general population are 50 points; thus, a score of <50 in either summary indicates an impaired QOL.

### 2.4 mFSSG

Kusano et al^[15]^ developed the FSSG as a questionnaire focusing on GERD They then further modified the FSSG to enable it to distinguish FD from nonerosive reflux disease (NERD) in patients without any organic disease, including reflux esophagitis (modified Los Angeles classification: Grade ≥A^[16]^) by adding 2 more questions.^[17]^ The mFSSG consists of 14 questions (Table 1). Seven questions are related to NERD; the other 7 questions are related to dyspepsia. The total score includes the scores for both the dyspepsia and NERD components. When the dyspepsia score is higher than the NERD score, the patient is considered to have dyspepsia. On the other hand, when the NERD score is higher than the dyspepsia score, the patient is considered to have NERD. If both scores were equal, the patient was diagnosed with either reflux or dyspepsia according to the field (either reflux or dyspepsia) of the highest scoring question. If the highest scores of both fields were equal, the patient was considered to have overlapping symptoms.

**Table 1:**
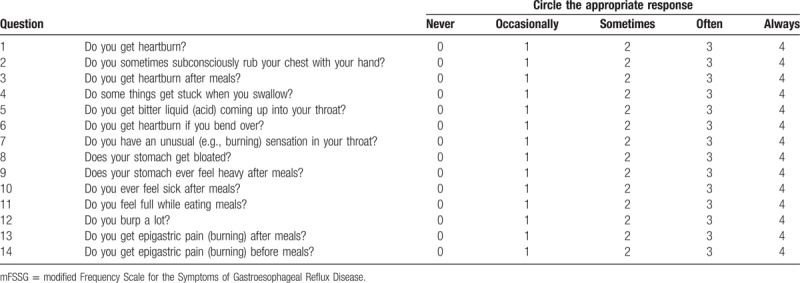
The mFSSG.

### 2.5 The eradication and its validation

All patients received regular arrangement of collecting venules therapy (Rabeprazole, 10 mg; Amoxicillin, 750 mg; Clarithromycin, 200 mg; b.i.d. for 7 days). ^13^C-UBT was performed to determine the efficacy of the Hp eradication treatment at 4 to 6 after treatment. The serum Hp IgG, pepsinogen I, II, levels and the pepsinogen I/II ratio were also measured at each point. Patients whose Hp IgG antibody titer was <10 U/mL and ≥10 U/mL were considered to be Hp negative and Hp positive, respectively.

### 2.6 ABC classification

The patients were divided to 4 groups based on the combination of the serum pepsinogen level determined by a pepsinogen test (PGT) and their Hp IgG antibody status. The most suitable cutoff point for prostaglandin (PG) positivity was reported to be a PG I concentration of <70 ng/mL and a PG I to PG II ratio of <3.0.^[18]^ The patients were classified into Groups A to D as follows: Group A, Hp IgG antibody negative and PG negative; Group B, Hp IgG antibody positive and PG negative; Group C, both Hp IgG antibody positive and PG positive; and Group D, Hp IgG antibody negative and PG positive. The number of patients in each of these groups was analyzed as a factor that was associated with the QOL.

### 2.7 Statistical analysis

The SF-8 score, mFSSG score, the HP IgG titer, the PG I level and the PG I/II ratio before and after the eradication of Hp were compared using a paired *t* test. The body-mass index was compared using the Mann–Whitney *U* test. The factors that contributed to the QOL were evaluated using a multiple logistic regression analysis. The other factors were compared by a chi-squared test or Fisher's exact test. The data were presented as the mean ± standard deviation. In all of the analyses, a *P* <0.05 was considered to indicate statistical significance. All of the statistical analyses were performed using the SAS software program (version 9.3; SAS Institute Inc., Cary, NC), or the SPSS for Windows software program (version 22.0; IBM, Tokyo, Japan).

## 3 Results

### 3.1 The participants and Hp infection

All 233 patients were diagnosed with “endoscopic atrophy of the gastric mucosa” by endoscopy using white light imaging, and were enrolled in the study. One hundred eighty-four of 233 patients were diagnosed with Hp infection according to the presence of Hp IgG antibodies. The background information of the 184 Hp-infected patients who were enrolled in the present study is shown in Table 2. The male/female ratio was 92/92. The mean PCS and MCS scores on the SF-8 were 48.7 (±6.2) and 50.3 (±5.7), respectively. The patients’ epigastric symptoms, as determined by the mFSSG, were categorized as follows: NERD, 29.9% (n = 55); dyspepsia, 37.5% (n = 69); overlapping symptoms, 10.3% (n = 19); asymptomatic, 22.3% (n = 41).

**Table 2:**
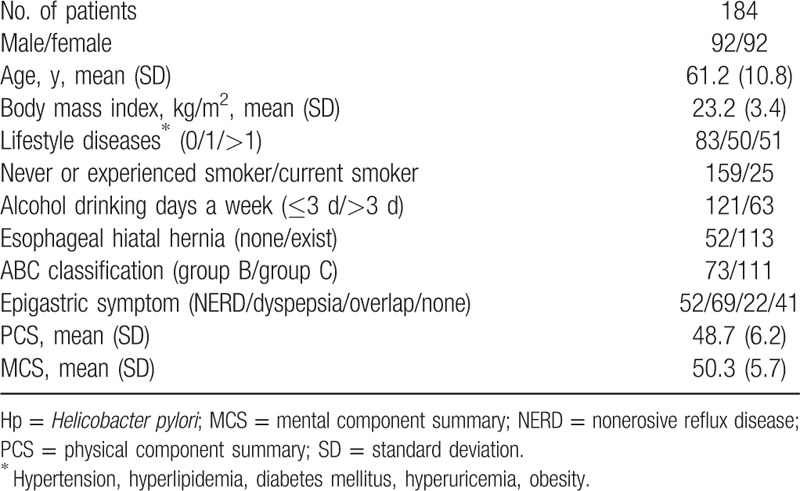
The background characteristics of the Hp-positive patients.

One hundred and sixty-five of the 184 patients underwent Hp eradication therapy, the validation of which was performed for all 165 patients (Fig. 1). All 165 Hp-infected patients underwent Hp eradication therapy, and validation was performed for all 165 patients. The rate of successful Hp eradication was 82.4% (136/165).

Finally, 160 out of 165 patients were eligible for analysis because the patients completed SF-8 questionnaires both before and after the eradication treatment.

### 3.2 The epigastric symptoms after the eradication of Hp

**Figure 2:**
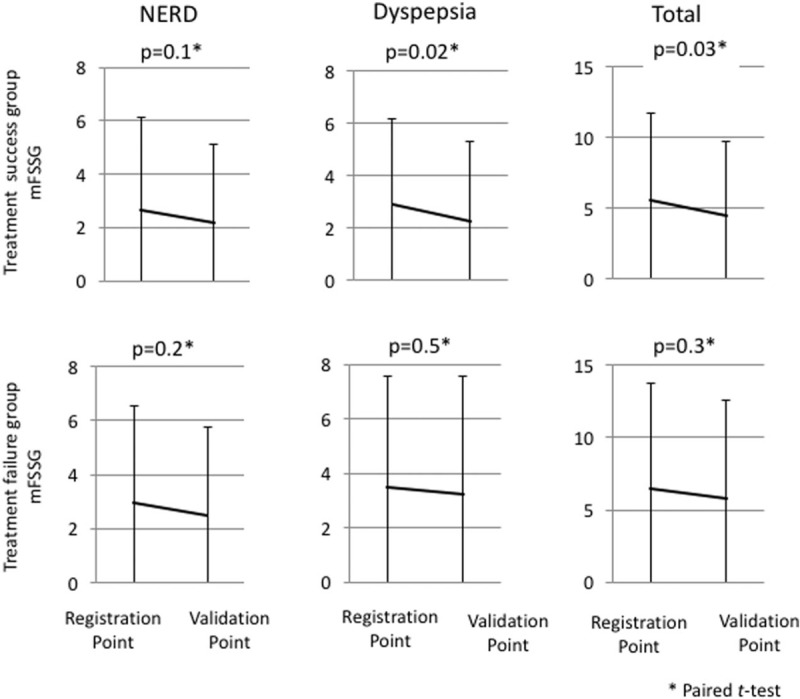
The upper 3 figures show the changes in the epigastric symptoms among the patients in the treatment success group (N = 133); the lower 3 figures show the changes in the epigastric symptoms in the treatment failure group (N = 27). With the exception of NERD, the epigastric symptoms improved in the treatment success group. None of the epigastric symptoms improved in the treatment failure group. mFSSG = modified Frequency Scale for the Symptoms of Gastroesophageal Reflux Disease; NERD = nonerosive reflux disease.

Of the 165 patients who underwent Hp eradication therapy, 160 were eligible for the mFSSG. Regarding the 127 patients who had epigastric symptoms as determined by the mFSSG, the details of the symptoms were as follows: NERD, 38% (n = 48); dyspepsia, 48% (n = 61); and overlapping symptoms, 14% (n = 18) in patients who underwent Hp eradication therapy. Dyspepsia and total epigastric symptoms were significantly improved in patients in whom Hp eradication was successful. In contrast, these symptoms did not significantly improve in the patients in whom Hp eradication treatment failed (Fig. 2).

### 3.3 The QOL after the eradication of Hp

**Figure 3:**
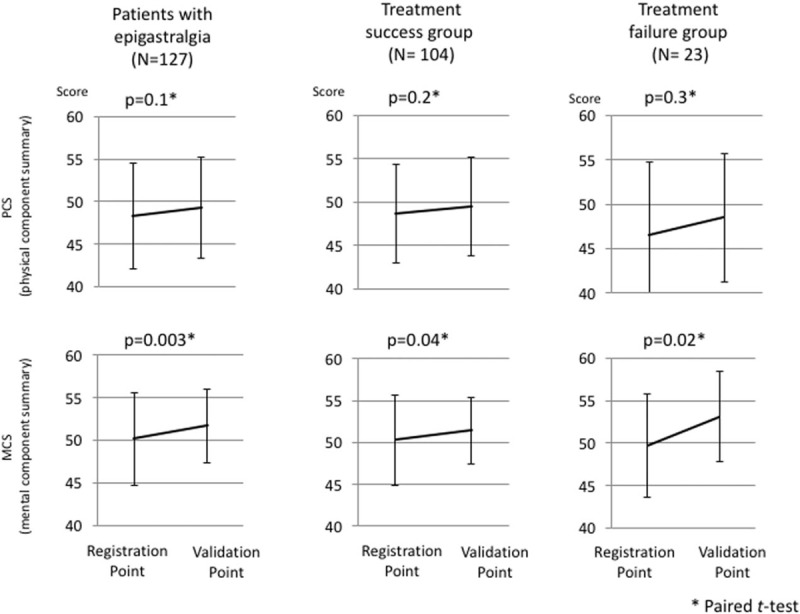
The change in the QOL following Hp eradication treatment in patients with epigastralgia (N = 127) and in the treatment success (N = 104) and treatment failure (N = 23) groups. Among total patients, both the PCS and MCS scores showed significant improvements after the eradication of Hp. In the treatment success group, although the PCS score was not improved after Hp eradication treatment, the MCS did show a significant improvement. In the treatment failure group, although the PCS score was not significantly improved after Hp eradication treatment, the MCS score did show a significant improvement. Hp = *Helicobacter pylori*; MCS = mental component summary; PCS = physical component summary.

Among the 165 patients who underwent Hp eradication therapy, 160 patients were eligible for the QOL analysis. The SF-8 questionnaire showed that both the PCS and MCS scores were significantly improved after Hp eradication therapy in all patients (PCS; *P* = 0.04, MCS; *P* = 0.004). In the 127 patients who had with epigastric symptoms, the mean PCS and MCS scores on the SF-8 were 48.3 (±6.2) and 50.2 (±5.5), respectively. The MCS scores were significantly improved after Hp eradication therapy in the treatment success as well as the treatment failure groups (Fig. 3).

### 3.4 The factors associated with an improvement in the QOL

We analyzed the factors associated with the QOL in patients whose QOL was improved and in those whose QOL showed no improvement (Tables 3 and 4). At the PCS evaluation, a low PCS score: PCS < 50 before the eradication of Hp was significantly associated with an improved PCS score on both the univariate and multivariate analyses (Table 3). The univariate analysis revealed that the patients in whom the MCS improved were younger and that the group included a larger number of people with an MCS value of <50 before the eradication of Hp (in comparison to those in whom the MCS worsened). The multivariate analysis revealed that an MCS value of <50 before the eradication of Hp was the only factor that contributed to an improvement in the MCS (Table 4). However, we did not indicate the factors that were associated with both the PCS and the MCS in this study (Table 5).

**Table 3:**
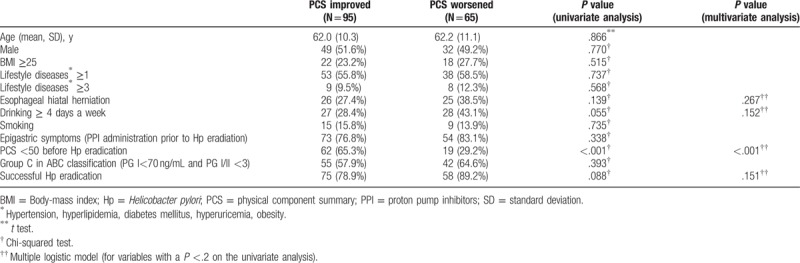
The analysis of the PCS before and after Hp eradication: groups presented PCS improved/PCS worsened.

**Table 4:**
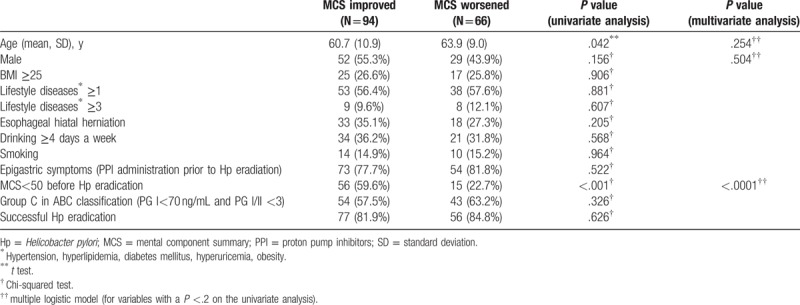
Analysis of the MCS before and after Hp eradication.

**Table 5:**
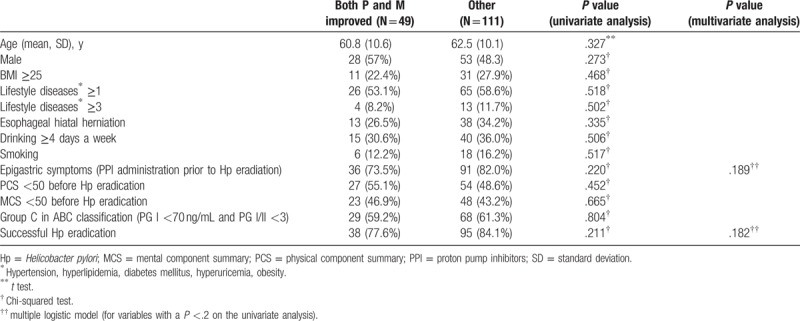
The analysis of both the PCS and MCS before and after Hp eradication.

### 3.5 The Hp IgG and PGT results after the eradication of Hp

**Figure 4:**
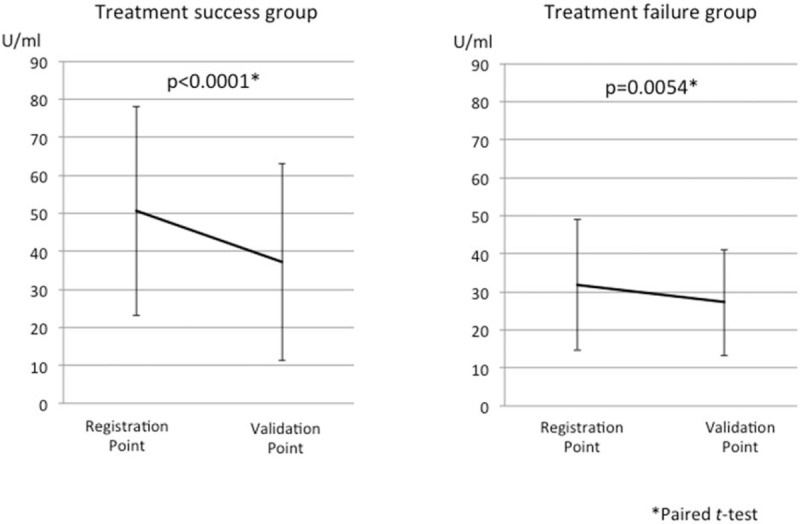
The change in the serum Hp IgG titers of the treatment success (N = 135) and treatment failure (N = 29) groups. Hp IgG antibody titer was significantly decreased in both the treatment success and treatment failure groups; however, the grade of the decrease was significantly higher in treatment success group. Hp = *Helicobacter pylori,* IgG = immunoglobulin G.

**Figure 5:**
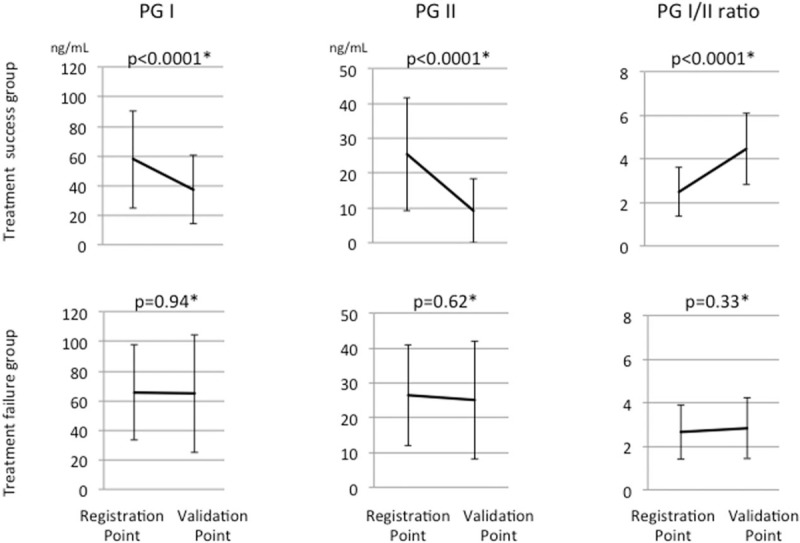
The 3 upper figures show the changes in the PGT results in the treatment success group (N = 131); the 3 lower figures show the changes in the PGT results in treatment failure group (N = 27). The PG I and PG II values and the PG I/II ratio were significantly improved in the treatment success group, but not in the treatment failure group. PGT = pepsinogen test.

The data of all 165 patients who underwent Hp eradication therapy were included in the analysis of the Hp IgG and PGT results after the eradication of Hp. Both the patients in whom Hp was successfully eradicated (the treatment success group; n = 136) and those in whom Hp eradication treatment failed (the treatment failure group; n = 29) showed a significant decrease in the Hp IgG titer after the eradication of Hp (Fig. 4). The PG I and PG II values significantly decreased, and the PG I/II ratio significantly increased in the patients in whom Hp eradication was successful. In contrast, there were no significant changes in these variables among the patients in whom Hp eradication treatment failed (Fig. 5).

## 4 Discussion

Although, all 233 patients with endoscopically diagnosed atrophic gastritis were included in this study, only 184 of the 233 patients were really Hp positive. Almost all of the Hp-negative patients were diagnosed with “antrum-predominant gastritis”. This result means that the endoscopic diagnosis of atrophic gastritis was made using only white light. It is particularly difficult to determine whether the gastric mucosa is normal or whether mild atrophy (i.e., “antrum-predominant gastritis”) is present using white light alone. According to Kyoto classification for atrophic gastritis, which was established in 2014, the color fading area of only the antrum is defined “no atrophy.”^[19]^ The identification of fading color in the antrum color area should not be used on its own to diagnose atrophic gastritis.

The QOL in all of the Hp-positive patients was improved after the eradication of Hp, regardless of the presence or absence of epigastric symptoms. In the present study, a lower QOL score before the eradication of Hp was associated with an improved QOL after Hp eradication treatment. Several other authors have reported that the epigastric symptoms and QOL of Hp-positive patients with GERD, FD, and peptic ulcers were significantly improved by the eradication of Hp.^[10,11,20]^ However, these previous studies only evaluated the symptoms and QOL in patients who succeeded with Hp eradication. In contrast, the present study evaluated all patients, regardless of the results of Hp eradication. Our study showed that the QOL of Hp patients without epigastric symptoms were also improved by the eradication of Hp. We found that Hp eradication therapy was effective in improving symptoms and the QOL, regardless of the outcome of the treatment.

It was reported that proinflammatory cytokines, including interleukin-1β (IL-1β), interleukin-6 (IL-6), and tumor necrosis factor-α (TNF-α), are involved in the etiologies of mental disorders.^[21]^ Furthermore, elevated Hp IgG levels were significantly associated with increased serum IL-6 levels among Hp-infected individuals.^[22]^ We measured the IL-1β concentration of preserved analyzable serum in some patients (N = 10); however, we noted no significant differences in the concentration between the Hp eradication success group and failure group (data not shown). The serum levels of several cytokines, including IL-6 or TNF-α, may be decreased after the eradication of Hp. As such, improvements in these levels can be expected in patients with mental disorders based on improvements in both the PCS and MCS scores. We hope to measure these cytokines in serum or gastric tissues in the near future.

The serum levels of both PG I and II were significantly decreased after the successful eradication of Hp, and the PGI/II ratio was significantly increased. These results might validate those of a previous report,^[23]^ and could mean that PGT was useful as an alternative method for validating the eradication of Hp. Interestingly, the Hp IgG titers of patients in whom Hp eradication failed also decreased after the eradication treatment, similar to the patients in whom Hp eradication was successful. A decrease in the Hp IgG titer means that the actual number of Hp bacterial bodies decreased;^[24]^ this could be why the QOL were improved after the eradication of Hp, irrespective of whether the eradication treatment was successful. On the other hand, we concluded that there was no relationship between the improvement in the PGT and that in the QOL in this study.

There present study is associated with some limitations. First, it involved a small number of participants and a short observation period. Second, the term “dyspepsia” was not strictly equivalent to “FD”. FD is diagnosed according to the Rome III criteria when patients satisfy the following 4 symptoms, “bothersome postprandial fullness,” “early satiation,” “epigastric pain,” and “epigastric burning,” without any structural disease. Furthermore, the criteria must be fulfilled for at least 3 months from the onset of symptoms, from at least 6 months prior to the diagnosis.^[25]^ As we did not take the period of symptoms into account in this study, we used the term “dyspepsia” rather than “FD”. Kinoshita et al^[26]^ reported that the ratio of with dyspepsia patients who fulfilled the definition for FD on the Rome III criteria was only 16.4%. However, under the Japanese insurance system, patients may present to a hospital soon after experiencing “dyspepsia,” thus, few patients had suffered from the symptom for more than 6 months. The patients with “dyspepsia” may be diagnosed with “FD” in actual clinical conditions.

There are some advantages to the present study. First, to date most studies have evaluated patients in whom Hp eradication was successful, whereas we evaluated all patients who underwent Hp eradication therapy in an intention-to-treat analysis. Second, the QOL of the Hp-positive patients with no symptoms had not been evaluated. Thus, this article is the first report to show the benefit of Hp eradication in all types of Hp patients from the point of view of QOL.

## 5 Conclusion

The eradication of Hp led to an improvement in the QOL, regardless of the success or failure of Hp eradication treatment or the presence or absence of previous epigastric symptoms, especially in patients who had an impaired QOL.
